# Editorial: Methodological advancements and improvements in structural biology

**DOI:** 10.3389/fmolb.2025.1625111

**Published:** 2025-06-24

**Authors:** Annalisa Pastore

**Affiliations:** ^1^ The Wohl Institute, King’s College London, London, United Kingdom; ^2^ Institute of Brain Sciences, Burlington Danes, The Hammersmith Hospital, London, United Kingdom; ^3^ Elettra Sicrotrone Trieste, Basovizza Trieste, Italy

**Keywords:** biophysics, X ray crystallography, cryo-EM, labelling for NMR studies, XFEL, small-angle X-ray scattering

While structural biology seems to be a mature field in which only few advancements might be possible, exciting breakthroughs continue to come up in several directions reassuring that the field is still active and vibrant. In the present Research Topics, six quite diversified studies are reported that propose new technical advancements in various different areas both at the software and data analysis level.

The work by Nam describes the intricacies of serial crystallography, a technique using X-ray free-electron lasers (XFEL) and synchrotron X-rays that has been introduced to study time-resolved molecular mechanisms through pump-and-probe experiments by an optical laser or a liquid application. One of the advantages of this technique is to be able to determine macromolecular structures at room temperature or near-physiological temperature with minimal radiation damage. The author specifically describes the meaning and usage of hit rate and indexing rate. The first refers to an important statistic that allows determination of the data acquisition efficiency and planning beamtime utilization in experiments. The second provides information about the crystal and data quality during data collection and processing. and is a statistic obtained by dividing the number of indexed images that match the input crystal information by the total number of hit images. The two parameters can be used to evaluate the sample quality, data collection strategy, and beamtime efficiency during data collection and processing. While still not widely used because not yet easily accessible, serial crystallography constitutes the future of structural biology to reconstruct macromolecular machines.

A second paper by the same author (Nam) a few months later discussed the effects of an imprecise molecular location in an ambiguous electron density map of the PDB-deposited structural model on the structure quality and how this could be improved using AI-predicted model structures. The author argues that by employing these model structures one could increase the precision of experimentally determined structures that will eventually be deposited in the PDB database and contribute to the advancement of fundamental scientific applications. However, the experimental and AI structures should be used complementarily.

Gomes et al. compared two popular optimization methods, ENSEMBLE and the Bayesian Maximum Entropy method, to fit with experimental data from nuclear magnetic resonance, small-angle X-ray scattering and single-molecule Förster resonance energy transfer. The analysis of the optimized ensembles considering several parameters such as secondary structure propensity, contact maps, and various types of interactions, revealed the importance of a physics-based generation of initial ensembles. Overall, the paper argued for the importance of generating increasingly accurate, reliable and experimentally validated ensembles. This is particularly important for disordered proteins.

The paper by Meng et al. comes from the Van Andel Institute (VAI), a private institution that, as many others, have made significant investments to establish a local cryo-EM core facility. The paper presents the implementation of this new mid-sized cryo-EM facility and discusses strategies to optimize routine operation to achieve higher performance and efficiency for single-particle cryo-EM. The whole project is described since its first conception including the initial planning, selection of equipment and development of IT solutions to improve data collection and analysis. The paper is an excellent guide for people who are planning to establish a non-standard local facility.

In the paper by Kim et al., the authors introduced Simple Scattering, an easy-to-use, open data repository for storing and sharing groups of correlated scattering profiles obtained from screening experiments of lipid nanoparticles (LNPs). These are particles important to safely and effectively deliver therapeutics. Simple Scattering collects small angle X-ray and neutron scattering data that can uniquely characterize LNPs. This provides a powerful tool that can be used for different chemical, biological and medical applications. The authors discussed the repository and its merits and analysed the possible limitations and future updates.

Finally, the paper by Kara et al. discusses an easy and straightforward NMR-based method to study protein-RNA interactions and to evaluate the binding of pairs of proteins to a single-stranded RNA under physiological conditions. The authors showed that they can readily incorporate a ^19^F atom on the ribose of nucleotides in any single strand RNA sequence. As a result, addition of an RNA binding protein to the RNA causes perturbation of the intensity of the ^19^F NMR signal changes when the ^19^F atom is located near the protein binding site. These studies can in principle be carried out in a nuclear extract that mimics the physiological environment in which these protein-ssRNA interactions occur. The authors demonstrated that a trifluoromethoxy group (-OCF_3_) incorporated in the 2′ribose position of single strand RNA sequences increases the sensitivity of the NMR signal.

These technical advancements demonstrate how active structural biology still is in the machine learning era and provide useful guidelines to improve the standing techniques.

